# Who uses height-adjustable desks? - Sociodemographic, health-related, and psycho-social variables of regular users

**DOI:** 10.1186/s12966-017-0480-4

**Published:** 2017-03-06

**Authors:** Birgit Wallmann-Sperlich, Tanja Bipp, Jens Bucksch, Ingo Froboese

**Affiliations:** 1 Institute for Sports Science, Julius-Maximilian University of Würzburg, Judenbühlweg 11, 97082 Würzburg, Germany; 2 Institute of Health Promotion and Clinical Movement Science, German Sport University Cologne, Am Sportpark Müngersdorf 6, 50933 Köln, Germany; 3 Work, Industrial, and Organizational Psychology, Julius Maximilian University of Würzburg, Röntgenring 10 - Room 330, 97070 Würzburg, Germany; 4 School of Public Health, Department of Prevention and Health Promotion, Bielefeld University, PO Box 100131, 33501 Bielefeld, Germany

**Keywords:** Cross-sectional, Desk-based, Height-adjustable desk, Occupational Sitting and Physical Activity Questionnaire, Office-workers, Sitting time, Correlates, Natural approach

## Abstract

**Background:**

Sit-to-stand height-adjustable desks (HAD) may promote workplace standing, as long as workers use them on a regular basis. The aim of this study was to investigate (i) how common HAD in German desk-based workers are, and how frequently HADs are used, (ii) to identify sociodemographic, health-related, and psycho-social variables of workday sitting including having a HAD, and (iii) to analyse sociodemographic, health-related, and psycho-social variables of users and non-users of HADs.

**Methods:**

A cross-sectional sample of 680 participants (51.9% men; 41.0 ± 13.1 years) in a desk-based occupation was interviewed by telephone about their occupational sitting and standing proportions, having and usage of a HAD, and answered questions concerning psycho-social variables of occupational sitting. The proportion of workday sitting was calculated for participants having an HAD (*n* = 108) and not-having an HAD (*n* = 573), as well as for regular users of HAD (*n* = 54), and irregular/non-users of HAD (*n* = 54). Linear regressions were conducted to calculate associations between socio-demographic, health-related, psychosocial variables and having/not having an HAD, and the proportion of workday sitting. Logistic regressions were executed to examine the association of mentioned variables and participants’ usage of HADs.

**Results:**

Sixteen percent report that they have an HAD, and 50% of these report regular use of HAD. Having an HAD is not a correlate of the proportion of workday sitting. Further analysis restricted to participants having available a HAD highlights that only the ‘perceived advantages of sitting less’ was significantly associated with HAD use in the fully adjusted model (OR 1.75 [1.09; 2.81], *p* < 0.05).

**Conclusions:**

The present findings indicate that accompanying behavioral action while providing an HAD is promising to increase the regular usage of HAD. Hence, future research needs to address the specificity of behavioral actions in order to enhance regular HAD use, and needs to give more fundamental insights into these associations.

## Background

Evidence is accumulating for the deleterious health effects of high levels of sitting. Current findings have shown that sitting time is consistently associated with an increased risk of all-cause mortality [1–4] and numerous other negative health conditions such as obesity [5], cardiovascular diseases [6, 7], and type 2 diabetes mellitus [6, 8] as well as various other metabolic risk factors [9]. Sedentary behaviours are highly prevalent in adults from high-income countries [10–12], suggesting that the majority of time awake is spent sitting. Occupational sitting time has been identified as one of the largest contributors to overall daily sitting time in white-collar workers [13, 14], so desk-based workers are particularly exposed to the health risks of prolonged sitting [15].

Consequently, reducing sitting time has recently been subject to health promotion in the workplace settings of desk-based workers [16]. Interventions aim to reduce sitting time during work [17], focusing especially on replacing sitting with standing, specifically through height-adjustable desks (HAD) [18]. A meta-analysis of 19 field-based trials and 19 laboratory investigations showed that the use of activity-permissive workstations, including HAD or treadmill desks etc. reduces sitting time in desk-based workers by around 77 min of sitting time/8-h workday (95% confidence interval = −120, −35 min) [19]. Further systematic reviews highlighted that the use of standing desks did not negatively influence work performance [19, 20] and productivity [21]. In addition, some studies underlined that breaking up sitting during work through standing bouts reduces the symptoms of fatigue and musculoskeletal discomfort [22]. Qualitative approaches showed a high acceptability and feasibility of HADs and complemented this field [23, 24]. However, these findings resulted from interventional trials that were specifically designed in a controlled research environment, aimed at reducing sitting times of desk-based workers through activity-permissive workstations.

The interest concerning HADs in research has increased in the last couple of years and the availability of HADs appears to be on a rise. However, to the best of our knowledge there is no information concerning the prevalence of HADs in non-interventional work settings. From a public health perspective it is even more interesting how frequently desk-based workers actually use HADs in the natural working setting, which helps to learn more about a potential population based effect. In more general terms, previous studies showed that higher education [14, 25, 26], younger age [25], and a perception of the advantages of sitting less at work [25] are associated with less occupational sitting times, showing contrary results for BMI [25, 27]. However, no study to date has investigated in more detail if having a HAD is a correlate of occupational sitting time in the natural, non-interventional environment.

Furthermore, socio-ecological frameworks have been proposed to understand and reduce sitting time [28]. Taking the introduction of HADs as an intervention at the environmental level of restructuring the office micro-architecture, it is also crucial to understand in more detail the sociodemographic, health-related and psychosocial correlates of frequent users vs. irregular or non-users of HADs to inform the development of and improve the effectiveness of HAD interventions for individual potential user groups.

Consequently, the aim of this study was to investigate (i) the availability of HADs in German desk-based workers and how frequently HADs are used in a non-interventional work setting, (ii) to identify sociodemographic, health-related and psycho-social correlates of occupational sitting including having a HAD, and (iii) to analyse sociodemographic, health-related and psycho-social correlates of users and non-users of HADs.

## Methods

### Study design

We conducted a cross-sectional questionnaire-based telephone study on health behaviours including questions about self-reported sitting time and physical activity (PA) in the workplace setting in Germany. Within this scope, the service research centre, Growth from Knowledge (GfK), in Nuremberg collected nationally representative data for the distribution of the German population between February and April 2016 as part of a computer-assisted telephone interview (CATI). Pre-tests were conducted in February 2016 during which the selected professional interviewers were trained in administering the computer-assisted standardised questionnaire. All study procedures were approved by the Ethics Committee of the German Sport University in Cologne.

### Sample

In total, 2830 representative residents (1386 men, 1444 women) from the 16 German federal states who were over 18 years of age (mean 50.4 ± 18.3) were interviewed. The sample was taken from the ADM Pool for Telephone Samples (ADM = Arbeitskreis der deutschen Markt- und Sozialforschungsinstitute – a study group of German market and social research institutions). The ADM pool is a precisely coordinated national sample, based on all possible telephone numbers, that forms the basis for population samples in the Federal Republic of Germany. The sample drawing was stratified according to age and gender, and the sample was weighted afterwards to the German population (year 2014) by federal state, residential density and household size according to the data from the National Federal Statistical Office. The overall response rate for the study sample was 13.5%. Based on the aims of the current study, we only included participants i) who were currently working, including participants in training and education, ii) who indicated that their work is predominantly desk-based 3) who answered questions concerning their sitting time during working hours, and 4) who were ≤65 years old. Because of these inclusion criteria and our data-cleaning process, we excluded data from respondents not working (*n* = 1228), not working predominantly desk-based (*n* = 868), due to missing values in one or all questions (*n* = 45; missing at random), due to their age (*n* = 9). Our final sample consisted of 680 participants (51.9% men; 41.0 ± 13.1 years).

### Measures

#### Availability of height-adjustable desks (HAD) and usage

Each participant was asked if their office desk was electronically height-adjustable with the answer options yes/no/don’t know. Participants who affirmed working at a HAD were asked if they use the height-adjustable function with answer options yes, regularly/irregularly/no. For further analyses we merged the answer options into two groups ‘regular use’ (*n* = 54) and ‘irregular/no use’ (*n* = 54). This item was developed specifically for this study and was pre-tested for face validity and participant comprehension.

#### Sitting time, PA and weekly working hours in the workplace setting

The Occupational Sitting and Physical Activity Questionnaire (OSPAQ) was used to assess sitting time and PA time in the office environment [29]. The OSPAQ is a validated instrument asking the participant to indicate the proportion of work time that she or he has spent sitting, standing, walking, and performing heavy labour on a typical workday in the last 7 days. All participants were also asked to report the number of hours they had worked in the last 7 days (weekly working hours) and the number of days they were at work. The OSPAQ shows satisfying test–retest reliability (ICC from 0.73 to 0.90) and moderate validity for estimating time spent sitting and standing at work compared to accelerometers (*r* = 0.65 and *r* = 0.49) [29, 30]. For further analysis we calculated the proportion of workday sitting.

#### Psycho-social variables

Psychosocial variables were adapted from previous studies [16, 25] that used psychosocial correlates for occupational sitting time of desk-based workers derived from existing theories of PA concepts in adults [31, 32]. Psychosocial variables included health knowledge about sitting [16], perceived organizational and social norms [16], perceived behavioural control [16], (dis)advantages of sitting less [25] and habit strength of sitting at the desk. Psychosocial variables were assessed using a 5-point Likert scale (1: strongly disagree to 5: strongly agree). Internal consistency and test-retest reliability are reported in detail [16] and range in these prior studies from Cronbach’s alpha 0.48–0.90 and test-retest reliability between 0.55 and 0.76. Regarding the scale of ‘knowledge’ Dunstan et al. [16] reported a Cronbach’s alpha below the acceptable value of <0.70. Based on this psychometric property and due to limited space within the questionnaire we used a single item solution to assess ‘knowledge’ which was ‘Sitting for most of the time at work is bad for my health’ for this scale. Furthermore, we selected items from more comprehensive scales. According to the construct of attitude we chose an item related to the affective part of attitude ‘for me to sit less at work is pleasant’ which seems an important part of a decisional balanced approach when weighing up advantages and disadvantages.

#### Socio-demographic variables

The demographic variables were self-reported age and gender. Additional socio-demographic variables included education that was classified into the following levels based on the German school system: ‘no school graduation’, ‘10 years of education’ (qualifies for training), ‘12 years of education’ (qualifies for technical college), ‘13 years of education’ (qualifies for university) and ‘first university degree or higher’.

#### Health-related variables

Body mass index was calculated through self-reported body weight and body height with the formula body weight (kg)/body height (meter)^2^. Subjective health status was measured through the question ‘how would you estimate your personal health status?’ with a 5-point Likert scale (graded excellent to very poor).

### Data analysis

We employed the data processing software PASW© (Version 23) for all statistical analyses. To address the first aim, descriptive statistics were calculated for the proportion of workday sitting by participants having HAD and also for those not-having HAD, as well as for regular users of HADs and irregular/non-users of HADs. For this purpose body mass index (<25 kg/m^2^/>25 kg/m^2^), subjective health status (excellent to good; fair to very poor) and psychosocial variables ((strongly) agree; neither agree nor disagree to (strongly) disagree) were dichotomized and the number of working hours per week were merged into three groups (≤20 working/hours/week; 20–40 working hours/week; >40 working hours/week).

For the second aim, referring to an ecological approach to sedentary behaviour [28], we used univariate and multiple linear regression analyses by the forced entry method to explore the associations between socio-demographic, health-related, psychosocial variables and having/not having a HAD (independent variables), and the proportion of workday sitting (dependant variables). The sociodemographic background variables were age (continuous variable), education level (five categories) and working hours per day (continuous variable). The health-related variables were body mass index (continuous variable) and subjective health status (five categories). Psychosocial variables had five categories and as an environmental variable we included having/not having a HAD (binary variable). To explore multicollinearity we used a bivariate correlation matrix for all independent variables and computed the variance inflation factors between all pairs of independent variables. We did not observe any problems with multicollinearity (variance inflation factor <2; bivariate correlation not higher than *r* = 0.35).

Binary univariate and multivariate logistic regressions analyses were conducted in order to examine the association between the independent socio-demographics, health-related and psychosocial variables of workplace sitting, and participants’ usage of HADs (binary dependent variable). We chose the forced entry method to explore the associations. Statistical significance was set at a level of *p* < 0.05.

## Results

### Availability and usage of HAD

Results show that 15.7% (*n* = 108) of the participants reported that they have an HAD at their workplace. They had daily proportions of workday sitting 71.3 ± 22.7 (see Table 1) and workday standing 12.6 ± 14.6 (data not shown) respectively. Of these participants, half (*n* = 54) reported a regular usage of the HAD (7.9%) with daily proportions of workday sitting 66.7 ± 25.0 and workday standing 15.0 ± 16.1 respectively. Irregular/non-users of HAD showed daily proportions of 75.8 ± 19.2% workday sitting and 10.3 ± 12.6% workday standing respectively.

**Table 1:** Descriptive characteristics of the proportion (SD) of workday sitting for sociodemographic variables of participants not having and having a height-adjustable desk (HAD) as well as for regular and irregular/non-users of HAD

	Proportion of workday sitting in % × ± SD (*n*)			
	Without HAD	With HAD	Regular Users HAD	Irregular/Non-Users HAD
All	73.3 ± 21.2 (573)	71.3 ± 22.7 (108)	66.7 ± 25.0 (54)	75.8 ± 19.2 (54)
Socio-demographic factors				
Gender				
Male	73.3 ± 20.3 (295)	71.2 ± 22.1 (58)	66.3 ± 23.5 (34)	78.3 ± 18.1 (24)
Female	73.3 ± 22.1 (278)	71.3 ± 23.6 (50)	67.5 ± 28.2 (20)	73.8 ± 20.2 (30)
Age Group				
18–29 years	75.1 ± 22.1 (159)	78.9 ± 13.3 (16)	83.0 ± 13.3 (10)	72.4 ± 11.4 (6)
30–45 years	75.7 ± 18.9 (193)	75.6 ± 17.7 (43)	69.9 ± 19.6 (24)	82.6 ± 12.4 (20)
46–65 years	70.0 ± 22.1 (221)	64.7 ± 27.3 (47)	54.7 ± 30.1 (20)	71.8 ± 23.3 (28)
Education				
‘no school graduation’	66.0 ± 23.5 (4)			
‘10 years of education’	56.5 ± 25.4 (29)	54.8 ± 9.6 (5)		54.8 ± 9.6 (5)
‘12 years of education’	69.1 ± 20.7 (149)	72.7 ± 21.9 (27)	74.5 ± 22.1 (15)	70.4 ± 22.4 (12)
‘13 years of education’	74.4 ± 22.3 (187)	66.7 ± 26.9 (40)	61.9 ± 30.4 (24)	74.0 ± 19.4 (16)
‘first university degree or higher’	78.0 ± 17.9 (203)	77.4 ± 17.2 (36)	66.9 ± 15.9 (15)	84.6 ± 14.3 (21)
Body Mass Index				
Healthy weight (BMI < 25 kg/m^2^)	73.9 ± 21.0 (356)	71.2 ± 25.4 (69)	65.9 ± 29.0 (34)	76.4 ± 20.4 (35)
Overweight–obese (BMI ≥ 25 kg/m2)	72.4 ± 21.5 (217)	71.4 ± 17.2 (38)	68.3 ± 16.6 (19)	74.6 ± 17.6 (19)
Working hours per week				
≤20 working hours/week	71.6 ± 27.0 (110)	77.6 ± 24.5 (11)	63.3 ± 44.3 (3)	82.5 ± 15.3 (9)
20–40 working hours/week	73.1 ± 18.3 (278)	65.6 ± 24.0 (64)	62.8 ± 27.0 (33)	68.5 ± 20.3 (31)
>40 working hours/week	74.7 ± 21.4 (185)	80.6 ± 14.8 (31)	74.9 ± 15.6 (17)	87.7 ± 10.2 (14)
Subjective health status^a^				
Excellent–good	74.0 ± 21.8 (417)	71.5 ± 20.9 (74)	67.2 ± 21.7 (40)	76.7 ± 19.1 (33)
Fair–very poor	71.5 ± 19.5 (156)	70.7 ± 26.4 (34)	65.3 ± 34.3 (13)	74.3 ± 19.9 (20)
Health Knowledge ‘Sitting for most of the time at work is bad for my health’^b^				
*n* (%) (strongly) agree	74.9 ± 20.8 (313)	67.5 ± 25.6 (61)	62.8 ± 26.5 (37)	74.6 ± 23.0 (24)
*n* (%) neither agree nor disagree, (strongly) disagree	71.4 ± 21.5 (259)	75.5 ± 16.7 (45)	73.0 ± 18.6 (15)	76.7 ± 15.9 (30)
Perceived organisational social norms ‘At my workplace nobody would mind if I chose to stand up while working at my desk’^b^				
*n* (%) (strongly) agree	73.3 ± 20.8 (388)	71.6 ± 22.8 (91)	67.3 ± 24.4 (46)	76.2 ± 20.2 (45)
*n* (%) neither agree nor disagree, (strongly) disagree	73.2 ± 22.3 (182)	65.8 ± 21.4 (15)	53.0 ± 25.9 (6)	73.7 ± 14.4 (9)
Perceived behavioural control ‘It is my choice whether I stand up or sit at my desk while at work’^b^				
*n* (%) (strongly) agree	71.8 ± 21.2 (314)	69.1 ± 22.8 (92)	64.5 ± 25.1 (48)	74.0 ± 19.1 (44)
*n* (%) neither agree nor disagree, (strongly) disagree	74.9 ± 21.1 (253)	83.6 ± 17.6 (12)	81.2 ± 18.0 (3)	84.4 ± 18.5 (9)
*Advantages of sitting less* ‘For me to sit less at work is pleasant’^b^				
*n* (%) (strongly) agree	70.4 ± 21.1 (264)	68.6 ± 21.5 (47)	59.8 ± 21.9 (26)	79.9 ± 15.0 (20)
*n* (%) neither agree nor disagree, (strongly) disagree	75.9 ± 20.7 (297)	72.2 ± 23.8 (57)	71.1 ± 27.4 (24)	73.0 ± 21.3 (33)
*Disadvantages of sitting less* ‘For me to sit less at work is not beneficial at all’^b^				
*n* (%) (strongly) agree	74.6 ± 21.2 (141)	77.1 ± 21.8 (26)	69.8 ± 25.4 (8)	80.4 ± 19.7 (18)
*n* (%) neither agree nor disagree, (strongly) disagree	72.9 ± 21.1 (418)	68.2 ± 22.6 (78)	63.9 ± 24.8 (42)	73.3 ± 18.9 (36)
*Habit* ‘I sit down at my desk without thinking about it’^b^				
*n* (%) (strongly) agree	76.0 ± 19.0 (406)	73.5 ± 22.3 (70)	66.0 ± 26.1 (31)	79.3 ± 16.9 (39)
*n* (%) neither agree nor disagree, (strongly) disagree	67.4 ± 24.3 (153)	65.3 ± 22.7 (35)	64.7 ± 23.5 (20)	66.1 ± 22.4 (14)

^a^Scored on a five-point scale (1–5) ranging from ‘excellent’ to ‘very poor’

^b^Scored on a five-point scale (1–5) ranging from ‘strongly disagree’ to ‘strongly agree’

### Correlates of workday sitting

Multivariate regression models showed that the availability of a HAD was not associated with the proportion of workday sitting. Age, perceived behavioural control and advantages of sitting less were negatively associated with workday sitting, so with increasing age, increasing perceived behavioural control and increasing perception of the advantages of sitting less, the proportion of workday sitting reduced. Education, BMI and habit were positively associated with workday sitting, showing that with a better education, a higher BMI and a stronger habit of ‘sitting down without thinking’ the proportion of workday sitting increased. The explained variance of this model was 12% (see Table 2).

**Table 2:** Results from univariate and multiple linear regressions on contribution of socio-demographic, health-related, environmental, and psycho-social variables on the proportion of workday sitting

	Univariate linear regression			Multiple linear Regression		
	Proportion of workday sitting (*n* = 637)			Proportion of workday sitting (*n* = 637)		
	B	SE B	β	B	SE B	β
Gender	0.02	1.65	0.00	1.10	1.76	0.03
Age	−0.17	0.06	−0.10**	−0.21	0.07	−0.13**
Education	5.08	0.87	0.22***	4.37	0.89	0.19***
Body Mass Index	0.19	0.19	0.04	0.53	0.22	0.10*
Working hours per week	0.08	0.05	0.06	0.07	0.05	0.06
Subjective health status	0.13	0.97	0.01	1.12	0.96	0.05
Height-adjustable desk (yes = 1/no = 2)	2.06	2.26	0.04	0.10	2.24	0.00
Health Knowledge ‘Sitting for most of the time at work is bad for my health’	0.47	0.64	0.03	0.50	0.67	0.03
Perceived organisational social norms ‘At my workplace nobody would mind if I chose to stand up while working at my desk’	−0.13	0.55	−0.01	0.31	0.61	0.02
Perceived behavioural control ‘It is my choice whether I stand up or sit at my desk while at work’	−1.29	0.49	−0.10**	−2.21	0.56	−0.17***
*Advantages of sitting less* ‘For me to sit less at work is pleasant’	−1.84	0.65	−0.11**	−1.99	0.68	−0.12**
*Disadvantages of sitting less* ‘For me to sit less at work is not beneficial at all’	0.45	0.63	0.03	0.80	0.62	0.05
*Habit* ‘I sit down at my desk without thinking about it’	3.11	0.61	0.20***	3.17	0.61	0.20***
				*Adj. R* ^*2*^ = 0.12		

B = unstandardized beta; SE B = standard error of beta; β = standardized beta

* = *p* < 0.05

** = *p* < 0.01

*** = *p* < 0.001

### Correlates of regular usage of height-adjustable desks

The only significant association within the multivariate model was between ‘perceived advantages of sitting less’ and regular use of the HAD (OR 1.75 [1.09; 2.81], *p* < 0.05), showing that participants with perceived advantages of sitting less were 1.75 times more likely to use their HAD. Univariate analyses showed that ‘health knowledge about sitting’ was significantly associated with regular use of the HAD (1.35 [1.00; 1.81], *p* < 0.05), meaning that participants with knowledge about ‘Sitting for most of the time at work is bad for my health’ were 1.35 times more likely to use their HAD (see Table 3).

**Table 3:** Results from logistic regression models predicting regular usage of height-adjustable desks

	Unadj. OR [95% CI]	Adj. OR [95% CI]
Gender	0.46 [0.21; 1.01]	0.39 [0.14; 1.12]
Age	0.97 [0.94; 1.01]	0.97 [0.93; 1.02]
Education	0.98 [0.63; 1.51]	0.86 [0.49; 1.53]
Body Mass Index	1.03 [0.93: 1.14]	1.09 [0.93; 1.28]
Working Hours per week	1.03 [1.00; 1.06]	1.00 [0.96; 1.05]
Subjective health status	0.78 [0.51; 1.19]	0.89 [0.50; 1.60]
Health Knowledge ‘Sitting for most of the time at work is bad for my health’	1.35 [1.00; 1.81]*	1.03 [0.69; 1.54]
Perceived organisational social norms ‘At my workplace nobody would mind if I chose to stand up while working at my desk’	1.23 [0.84; 1.81]	0.87 [0.51; 0.1.50]
Perceived behavioural control ‘It is my choice whether I stand up or sit at my desk while at work’	1.25 [0.82; 1.91]	1.40 [0.80; 2.45]
*Advantages of sitting less* ‘For me to sit less at work is pleasant’	1.69 [1.17; 2.45]**	1.75 [1.09; 2.81]*
*Disadvantages of sitting less* ‘For me to sit less at work is not beneficial at all’	0.75 [0.55; 1.01]	0.68 [0.45; 1.05]
*Habit* ‘I sit down at my desk without thinking about it’	0.93 [0.71; 1.22]	0.98 [0.67; 1.43]
		R^2^ = 0.21 (Cox & Snell), 0.28 (Nagelkerke). Model Chi-Square 24.04

* = *p* < 0.05

** = *p* < 0.01

## Discussion

The results of this study showed that about 16% of the participants with desk based occupations had a HAD available for potential use. However, only half of them used it regularly. Our results showed no association between having an HAD and sitting time. Further analyses restricted to participants having available a HAD highlighted that the only psychosocial factor that was significantly and positively associated with regular HAD use was ‘perceived advantages of sitting less’.

### Prevalence of workday sitting and HAD use

The reported proportion of workday sitting was in line with a recent Australian study [33]. Compared to studies which objectively measured occupational sitting time our prevalence was lower [27, 34, 35] and may be underestimated, but there is not much variance. The number of available HADs showed that the issue of reducing sitting time and replacing it with standing seems to have reached the office employers to some extent. However, this figure also shows a high potential to increase the availability of HADs in Germany. Further, we had no data about the reasons for the presence of HADs in the working environment. For example, employers in Germany have to provide HADs if any health-related problems like muscular-skeletal or back pain exist which could be a factor of the current prevalence as opposed to concern for the detrimental effect of time spent sitting during work.

Our data on regular usage of HADs, which was only present in half of the participants, were in contrast to the results of previous qualitative studies. Although previous studies showed that there are different patterns of usage of the HAD [24], all study participants made use of the new workstations [23]. Surprisingly, the proportion of sitting in the youngest age group of regular HAD users was higher in its tendency than irregular users, which is difficult to explain and needs further clarification in future studies. However, to the best of our knowledge we could not identify any comparable numbers for availability and usage of HAD within a natural and non-interventional setting. Collecting this information without a direct link to the conduction of a workplace intervention can give insight into the field of the office environment under real-life conditions, so that workplace health promotions can benefit.

### Association between workday sitting and availability of HAD

Obviously, our results from regression models showed no association between the availability of an HAD and the proportion of workday sitting, which can be partly explained through the infrequent use of the HAD by participants who have one available. Our results stand in contrast to results from intervention studies which showed that activity-permissive workstations reduced sitting time [19, 23, 35–38]. However, in most of these studies, a restructure of the office environment was accompanied by intervention strategies on a behavioural and organisational level [35, 36, 38] such as increasing health-related knowledge about sitting, or using managers as active champions. A randomized three-month trial comparing HAD-only interventions against a multi-component approach [39] produced encouraging results, showing an approximate threefold greater reduction of sitting time in the multi-component approach, including support for the individual and at the organisational level, compared to the HAD-only intervention group. Results from qualitative studies showed that participants were highly motivated, and curious to try the sit-stand workstations, and the interest in potential health benefits were the given reasons for initial HAD use [23, 24]. In our study we did not have any information about possible accompanying action such as behavioural measures, e.g., health-related knowledge concerning sitting, or procedures on the organisational level when providing the HAD. However, our results suggest that the provision of HAD in workplaces is not sufficient and effective alone to justify the cost of providing HADs on a larger scale.

### Sociodemographic, health-related and psycho-social correlates of workday sitting and HAD use

Concerning socio-demographic and health-related correlates, our results replicate previous studies; especially younger [14, 25], higher educated [14, 25, 40] and office workers with high BMIs [25] are at particular risk of high occupational sitting times. These findings are useful in identifying special target groups for future interventions that are aimed at reducing sitting time at work.

In addition our results showed that a strong ‘perceived behavioural control’, ‘perceived advantages of sitting less’ as well as a weak formed habit of ‘sitting down at my desk without thinking about it’ were important psycho-social variables that are associated with a reduced proportion of sitting time. This clearly underpins the fact that individual level variables are of importance in explaining occupational sitting. The importance of the psychological construct of behavioural beliefs, as asked for by perceiving the advantages of sitting less, has been shown before as a correlate of occupational sitting time [25].

From an interventional point of view this highlights the necessity to elicit the behavioural beliefs [41] about sitting and standing of desk-based workers, and accordingly develop persuasive communication strategies that provide information, and increase the perception of the advantages of reducing sitting for desk-based workers.

Further, the construct of habit strength seems to be an important correlate. Making the use of HADs a habit should be the major goal of interventions, as habits are stable behaviour changes that are largely independent of consciousness processing. Indeed, promoting HAD use as a habit needs a consistent and encouraging environment for standing, using HADs more often, and it needs a high degree of repetition [42]. Provision of HADs accompanied by regular prompting, and an initial promotion of implementation intentions might be a promising strategy in the workplace environment [41]. However, research into the psychosocial variables of occupational sitting time, and developing intervention strategies are both rare, and should be addressed in the future.

As discussed earlier our in depth analyses which were restricted to surveyed participants who had access to a HAD showed that for the regular use of an HAD the most important correlate was apparently the ‘perceived advantages of sitting less’. Consequently, when providing an HAD, the personal anticipation of positive consequences must be served. Possible accompanying action could be information about positive health effects that are associated with the reduction of occupational sitting time [3, 15, 43], addressing perceived barriers, such as working in an open plan office and disturbing colleagues’ privacy while standing [24], issues with the design of the HAD [24], or fear of losing productivity [23]. A prerequisite for developing specific interventions is to understand the most relevant behavioural beliefs so that they can be addressed (see above).

### Limitations and strengths

Strengths of this study include the initial reasonably large sample size and the natural setting/non-interventional perspective to investigate the availability and usage of HADs in the “real” office environment setting, which is unique.

Nevertheless, the sample size drops down in further analyses investigating correlates for regular usage of HADs. Future studies should aim for sufficiently powered larger sample sizes in order to investigate potential correlates of regular HAD use. The low response rate of 13.5% is a further limitation. This could have potentially been a result of the overall mean length of this general health survey (approx. 22.5 min). Comparing our study to other surveys [44], the present response rate still seems acceptable. Furthermore, we see future potential in defining terms such as ‘predominantly desk-based’ (e.g., >50% of work is desk-based), and ‘regular use of HAD’ e.g., ‘every week’ or ‘several times a day’ etc. more clearly in order to reduce bias and also to ask for the typical duration of the standing period. This would be of further importance to future sedentary behaviour research in this field. Beyond this, interviewers should explicitly ask if the HAD function is used to switch between sitting and standing positions.

Our information on availability of HAD as well as on sitting time was assessed by self-report. Consequently, our results might have been biased, owing to mis-classifications or social desirability bias. Future research should capture information on the availability of HADs through field surveys, and use both objective and subjective assessments of sitting time in order to collect important domain- and behaviour-specific sitting time information, and also to objectively measure total occupational sitting times as well as patterns of sitting [45]. Moreover, as mentioned above we did not assess any information about accompanying actions while providing an HAD, so we can only assume that they were limited, e.g., regarding HAD instructions. This is essential information for future cross-sectional designs in the natural setting. Furthermore, we did not ask the current reason for the presence of a HAD, which might also be an important confounding factor. Finally, we should keep in mind that the study was cross-sectional, thus we cannot infer causality.

## Conclusion

The present study gives initial insight into the availability and usage of HADs, the association of HADs on occupational sitting time in desk-based workers, and on sociodemographic, health-related and psycho-social correlates of users and non-users of HADs through a cross-sectional study approach. The findings showed that 16% of the participants have a HAD for potential use, but only half of them regularly use it. Furthermore, our findings suggest that the availability of an HAD is not associated with occupational sitting time and that ‘perceived personal advantages of sitting less’ can increase the chances of HADs being used frequently. These findings propose that accompanying behavioral action that especially addresses the advantages and pleasantness of sitting less during work, through information strategies, through increasing the awareness of perception while using HADs, as well as through initial promotion of implementation intentions to make the use of HADs a habit, is promising in terms of increasing the regular usage of HADs. Hence, future research needs to address the specificity of behavioral actions in order to enhance regular HAD use, and to provide more fundamental insights into these associations.

## Abbreviations

HAD: Height-adjustable desk
OSPAQ: Occupational Sitting and Physical Activity Questionnaire
PA: Physical activity
